# Recent advances in pre-clinical mouse models of COPD

**DOI:** 10.1042/CS20130182

**Published:** 2013-10-14

**Authors:** Ross Vlahos, Steven Bozinovski

**Affiliations:** *Lung Health Research Centre, Department of Pharmacology, University of Melbourne, Parkville, VIC 3010, Australia

**Keywords:** acute exacerbations of chronic obstructive pulmonary disease (AECOPD), chronic obstructive pulmonary disease (COPD), emphysema, inflammation, skeletal muscle wasting, smoking, AECOPD, acute exacerbations of COPD, BAL, bronchoalveolar lavage, BALF, BAL fluid, COPD, chronic obstructive pulmonary disease, GM-CSF, granulocyte/macrophage colony-stimulating factor, GOLD, Global initiative on chronic Obstructive Lung Disease, Gpx, glutathione peroxidase, HDAC, histone deacetylation, IL, interleukin, LTB_4_, leukotriene B_4_, MAPK, mitogen-activated protein kinase, MCP-1, monocyte chemotactic protein-1, MMP, matrix metalloproteinase, NE, neutrophil elastase, NF-κB, nuclear factor κB, Nrf2, nuclear erythroid-related factor 2, O_2_^•−^, superoxide radical, ONOO^−^, peroxynitrite, PDE, phosphodiesterase, PI3K, phosphoinositide 3-kinase, ROS, reactive oxygen species, RV, rhinovirus, SLPI, secretory leucocyte protease inhibitor, SOD, superoxide dismutase, TGF-β, transforming growth factor-β, TIMP, tissue inhibitor of metalloproteinases, TNF-α, tumour necrosis factor-α, *V*/*Q*, ventilation/perfusion

## Abstract

COPD (chronic obstructive pulmonary disease) is a major incurable global health burden and will become the third largest cause of death in the world by 2020. It is currently believed that an exaggerated inflammatory response to inhaled irritants, in particular cigarette smoke, causes progressive airflow limitation. This inflammation, where macrophages, neutrophils and T-cells are prominent, leads to oxidative stress, emphysema, small airways fibrosis and mucus hypersecretion. The mechanisms and mediators that drive the induction and progression of chronic inflammation, emphysema and altered lung function are poorly understood. Current treatments have limited efficacy in inhibiting chronic inflammation, do not reverse the pathology of disease and fail to modify the factors that initiate and drive the long-term progression of disease. Therefore there is a clear need for new therapies that can prevent the induction and progression of COPD. Animal modelling systems that accurately reflect disease pathophysiology continue to be essential to the development of new therapies. The present review highlights some of the mouse models used to define the cellular, molecular and pathological consequences of cigarette smoke exposure and whether they can be used to predict the efficacy of new therapeutics for COPD.

## INTRODUCTION

**Figure 1:**
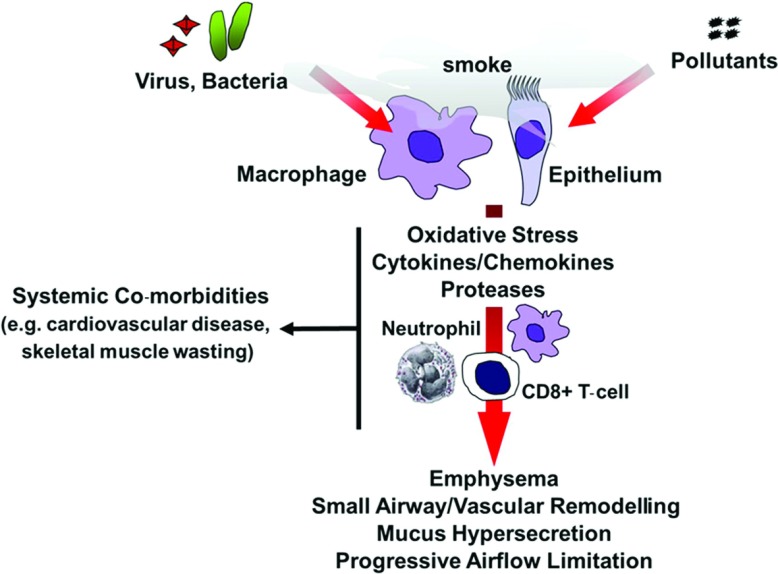
General model of cigarette smoke-induced lung inflammation and damage Cigarette smoke acts on alveolar macrophages and epithelium to induce the production of various cytokines and chemokines that serve to perpetuate the inflammatory response through the recruitment of peripheral blood monocytes, neutrophils and CD8^+^ T-cells into the airways. Activated macrophages and neutrophils release proteases, which cause tissue destruction and emphysema. Increased oxidative stress causes lung inflammation and cell and tissue injury. COPD patients are susceptible to viral and bacterial infections, which may amplify lung inflammation and cause a rapid decline in lung function.

COPD (chronic obstructive pulmonary disease) is a major global health problem and has been predicted to become the third largest cause of death in the world by 2020 [1]. Cigarette smoking is the major cause of COPD and accounts for more than 95% of cases in industrialized countries [2], but other environmental pollutants are important causes in developing countries [3]. COPD is ‘a disease state characterized by airflow limitation that is not fully reversible’. The airflow limitation is usually progressive and associated with an abnormal inflammatory response of lungs to noxious particles and gases [4]. COPD encompasses chronic obstructive bronchiolitis with fibrosis and obstruction of small airways, and emphysema with enlargement of airspaces and destruction of lung parenchyma, loss of lung elasticity, and closure of small airways (Figure 1). Most patients with COPD have all three pathological conditions (chronic obstructive bronchiolitis, emphysema and mucus plugging), but the relative extent of emphysema and obstructive bronchiolitis within individual patients can vary. The severity of COPD is currently classified according to GOLD (Global initiative on chronic Obstructive Lung Disease)/WHO (World Health Organization) criteria on a 0–4 scale based largely on deterioration of lung function and symptoms. Alternative scales, such as the BODE Index (Body mass index, airflow Obstruction, Dyspnoea and Exercise capacity index in chronic obstructive pulmonary disease), have been proposed and may offer a more comprehensive measure of disability as they also include systemic wasting indices [5]. As the disease worsens, e.g. Gold 2+, patients also experience progressively more frequent and severe exacerbations, which are due in greatest part to bacterial and viral chest infections, as well as pollutants [6–8]. The majority of the total health costs of COPD are associated with these acute exacerbations (AECOPD). Patients are also increasingly disabled by disease co-morbidities, especially skeletal muscle wasting and cardiovascular diseases, which further reduce their quality of life [9].

Inflammation Chronic obstructive pulmonary disease

The tragedy of COPD research is that the cause of the disease and the best way to prevent its incidence and progression are already known. As such, COPD is largely preventable and its progression can be largely halted, at least in the early stages, by smoking cessation. Owing to the addictive properties of tobacco, patients with early COPD are very frequently unable to quit smoking, even with intensive interventions. In addition, it is known that once inflammation is established in COPD it persists when smoking has stopped and is present even decades later. This inflammation responds marginally or not at all to current anti-inflammatory drugs, including high-dose inhaled glucocorticosteroids [10]. There is therefore a need for better and more effective treatments for COPD, AECOPD and COPD co-morbidities. Given that cigarette smoke is the major cause of COPD, ‘smoking mouse’ models that accurately reflect disease pathophysiology have been developed and have made rapid progress in identifying candidate pathogenic mechanisms and new therapies (reviewed in [11–17]).

## INFLAMMATORY CELLS AND MEDIATORS INVOLVED IN THE PATHOPHYSIOLOGY OF COPD

A variety of cell types are involved in the pathophysiology of COPD, including macrophages, neutrophils and T-cells. The airways, lung parenchyma, BALF [BAL (bronchoalveolar lavage) fluid] and sputum of patients with COPD have elevated levels of macrophages compared with normal smokers [18–20]. There is a correlation between macrophage numbers in the airways and the severity of COPD [21]. Macrophages release inflammatory mediators, including TNF-α (tumour necrosis factor-α), MCP-1 (monocyte chemotactic protein-1), ROS (reactive oxygen species) and neutrophil chemotactic factors, such as LTB_4_ (leukotriene B_4_) and IL (interleukin)-8, in response to cigarette smoke and other irritants. The sputum of COPD patients has increased levels of LTB_4_, IL-8 and TNF-α [19]. Alveolar macrophages also secrete a variety of elastolytic enzymes, including MMP (matrix metalloproteinase)-2, MMP-9, MMP-12, and cathepsin K, L and S, which destroy lung parenchyma [2]. Histological and bronchial biopsy studies show that patients with COPD have an increased number of neutrophils [20,21]. In addition, a marked increase in neutrophils has been observed in BALF and sputum of COPD patients [18,19]. Neutrophil numbers in bronchial biopsies and induced sputum are correlated with COPD disease severity [19,21] and with the rate of decline in lung function [22]. Neutrophils secrete serine proteases, including NE (neutrophil elastase), cathepsin G and proteinase-3, as well as MMP-8 and MMP-9, which contribute to lung destruction [2]. Neutrophils migrate into the respiratory tract under the direction of neutrophil chemotactic factors, which include IL-8 [2]. Neutrophil survival in the respiratory tract may be increased by cytokines, such as GM-CSF (granulocyte/macrophage colony-stimulating factor) and G-CSF (granulocyte colony-stimulating factor) [2]. Patients with COPD have an increased number of T-cells as shown by histological and bronchial biopsy studies [20,21,23]. Smokers with COPD have more CD8^+^ T-cells in central airways, peripheral airways, pulmonary arteries and lymph nodes than smokers without COPD [24]. In addition, smokers with severe COPD have increased levels of both CD4^+^ and CD8^+^ T-cells in the airway wall [24]. CD8^+^ T-cells are important for resolution of virus infections, but excessive recruitment results in inflammatory damage to lungs and a decline in lung function [23,25]. Hogg et al. [26] have investigated the nature of the inflammatory response in the small airways (less than 2 mm in internal diameter) in patients with COPD. In that study, progression of COPD from GOLD stage 0 to 4 was associated with an increase in the volume of the airway wall tissue (due to an increase in epithelium, lamina propia, muscle and adventitia) and the accumulation of inflammatory mucous exudates in the lumen of the small airways. The percentage of small airways that contained neutrophils, macrophages, CD4^+^ cells, CD8^+^ cells, B-cells and lymphoid aggregates containing follicles also increased as COPD progressed [26].

## PROTEASES IN COPD

Proteases regulate lung inflammation via the production of cytokines and chemokines and ultimately destroy the extracellular matrix (particularly elastin) of lung parenchyma to produce emphysema [27]. The major proteases involved in COPD include NE and various MMPs, although other serine proteases such as cysteine proteases and proteinase 3, have been implicated [2,27,28]. Patients with emphysema have an increase in BALF concentrations and macrophage expression of MMP-1, MMP-2 and MMP-9 [29,30] and activity of MMP-9 in the lung parenchyma [31]. Alveolar macrophages from patients with COPD express more MMP-9 and have enhanced elastolytic activity than those from normal smokers or non-smokers [29,32]. It is well documented that patients with an inherited deficiency of α_1_-antitrypsin, the endogenous inhibitor of NE, are at increased risk of emphysema. All of these proteolytic enzymes are counteracted by anti-proteases and it is believed that there is an imbalance between proteases and endogenous anti-proteases which normally protect against protease-mediated effects. The major inhibitors of serine proteases are α_1_-antitrypsin in lung parenchyma and airway epithelium-derived SLPI (secretory leucocyte protease inhibitor) in the airways. At least four TIMPs (tissue inhibitors of metalloproteinases; TIMP1–TIMP4) counteract MMPs, although there is conflicting evidence for their role in COPD [32–35].

## OXIDATIVE STRESS IN COPD

Oxidative stress plays an important role in COPD given the increased oxidant burden in smokers. The increased oxidant burden results from the fact that cigarette smoke contains over 4700 chemical compounds and more than 10^15^ oxidants/free radicals and that many of these oxidants are relatively long-lived [36]. These oxidants give rise to ROS by inflammatory and epithelial cells within the lung as part of an inflammatory-immune response towards a pathogen or irritant. Activation of Nox2 (NADPH oxidase 2) on macrophages, neutrophils and epithelium by cigarette smoke generates O_2_^•−^ (superoxide radical), which can then either react with NO to form highly reactive ONOO^−^ (peroxynitrite) molecules or alternatively be rapidly converted into damaging H_2_O_2_ under the influence of SOD (superoxide dismutase) [37–39]. This in turn can result in the non-enzymatic production of damaging ^•^OH (hydroxyl radical) from H_2_O_2_ in the presence of Fe^2+^. Polymorphisms in extracellular SOD have been associated with reduced lung function and susceptibility to COPD [40]. Gpxs (glutathione peroxidases) and catalase serve to convert toxic H_2_O_2_ into water and oxygen. The ROS O_2_^•−^, ONOO^−^, H_2_O_2_ and ^•^OH then trigger inflammation, DNA damage, protein denaturation and lipid peroxidation [41]. In addition, neutrophils are a rich cellular source of ROS and neutrophil-derived myeloperoxidase metabolizes H_2_O_2_ in the presence of Cl^−^ to generate the strong oxidant hypochlorous acid [41]. The net effect of all this ROS activity is that smokers and patients with COPD have higher levels of exhaled ROS than non-smokers, which are increased further in exacerbations [42].

## MODELLING COPD IN MICE

COPD is a heterogenous disorder consisting of lung inflammation, chronic obstructive bronchiolitis, mucus plugging and emphysema. Animal models are important in determining the underlying mechanisms of COPD as they address questions involving integrated whole-body responses. To date, many species have been used, including rodents, dogs, guinea-pigs, monkeys and sheep (reviewed in [14–16,43–45]). Mice offer the greatest ability to investigate the pathogenic pathways of disease given the low cost, the ability to produce animals with genetic modifications that shed light on specific processes within COPD, the plethora of information about the mouse genome, the abundance of antibody probes, and the availability of numerous naturally occurring mouse strains with different reactions to smoke. Although mice and humans share many basic physiological processes, animal models should be one component of the process for studying human disease and should be integrated with human *in vitro* and in *vivo* studies.

In mice, features characteristic of human COPD can be modelled by exogenous administration of proteases, chemicals, particulates and exposure to cigarette smoke [11–16]. Given that cigarette smoke is the major cause of COPD, many groups are investigating the cellular and molecular responses triggered by cigarette smoke [14–16,43–67]. A survey of this work shows there are basically two types of cigarette smoke exposures: nose only and whole-body exposure. Exact comparisons of findings from various groups are difficult because different types of cigarettes (reference compared with commercial), doses of cigarettes, instruments, exposure protocols and a wide variety of mouse strains are used. However, regardless of the method of exposure, many of the hallmark features of human COPD, namely (i) chronic lung inflammation (i.e. accumulation of macrophages, neutrophils and lymphocytes), (ii) impaired lung function; (iii) emphysema; (iv) mucus hypersecretion; (v) small airway wall thickening and remodelling (increased matrix components, inflammatory cells, and goblet cell metaplasia in the airway wall with luminal narrowing, distortion, and obstruction by mucus); (vi) vascular remodelling; (vii) lymphoid aggregates; and (viii) pulmonary hypertension, can be mimicked in the ‘smoking mouse model’ (Table 1). It must be noted though that there is no one perfect animal model of COPD that replicates all of the characteristic features of human disease. To complicate matters even more, not even one human would meet all of the above criteria because there is considerable human to human variation in the pattern of COPD. In addition, no good animal model of chronic bronchitis is available since the definition is clinical and the pathological changes in humans do not reliably separate bronchitics from non-bronchitics. Therefore the animal model should be chosen that is appropriate to the question being asked.

**Table 1:** Features of COPD that can/cannot be modelled in cigarette smoke-exposed mice

Can be modelled	Cannot be modelled
BALF/lung inflammation, including neutrophilia, accumulation of macrophages and T-cells, and lymphoid aggregates/follicles Increase in BALF/lung inflammatory mediators, including cytokines, chemokines and proteases	Chronic bronchitisSevere disabling disease observed in GOLD stage 3 to 4
Increased oxidative stress	
Emphysema	
Small airway and vascular remodelling	
Pulmonary hypertension	
Mucus hypersecretion	
Impaired lung function	
Systemic co-morbidities (e.g. cardiac dysfunction and peripheral skeletal muscle wasting)	
Increased BALF/lung inflammation in response to respiratory pathogens associated with infectious exacerbations	
Persistence of BALF/lung inflammation following smoking cessation	

Given the laborious nature of chronic cigarette smoke-exposure protocols, many groups have used acute cigarette smoke-exposure protocols to explore the mediators and mechanisms that drive cigarette smoke-induced lung inflammation and damage. This approach is often used as a ‘high-throughput screen’ to explore mechanisms and test drugs before being taken in to more chronic models of COPD. Churg et al. [48] have described an acute murine model of exposure to smoke from four cigarettes over 1 h. They observed that the resulting inflammation was associated with activation of the transcription factor NF-κB (nuclear factor κB) and proposed that TNF-α, processed by MMP-12, was largely responsible for the ensuing responses, especially up-regulation of the vascular adhesion molecule E-selectin [48]. These studies complemented their earlier work in a single exposure acute model, implicating TNF-α-dependent neutrophil recruitment in the protease-dependent breakdown of connective tissue, a precursor of emphysema, and in part reconciled conflicting data on the relative importance of neutrophil- compared with macrophage-dependent disease processes by providing a rational link between these cells [49]. We have shown that Balb/C mice exposed to cigarette smoke (whole-body exposure system) generated from Winfield Red cigarettes (16 mg or less of tar, 1.2 mg or less of nicotine and 15 mg or less of CO) for 4 days had an increase in BALF macrophages, neutrophils and protease expression. Using qPCR (quantitative real-time PCR), we have shown [52] that whole-lung extracts contained increased levels of cytokines, chemokines and neutrophil survival cytokines. In addition, there was a significant increase in the DNA binding of regulatory transcription factors [52]. We have supported these sub-chronic protocols with long-term studies and found that mice exposed to 3–6 months of cigarette smoke develop emphysema and a characteristic lung inflammation phenotype reminiscent of human COPD. Thus acute responses to smoke exposure may be useful as a predictor of the development of emphysema, and may be a useful screen by which to identify therapeutic targets.

The role of proteases in the development of COPD has been investigated in a number of species. Studies in gene-deficient mice have shown that emphysema induced by chronic cigarette smoke exposure is prevented in MMP-12^−/−^ mice [68]. In addition, mice in which the integrin αvβ6 is deleted fail to activate TGF-β (transforming growth factor-β) and develop age-related emphysema which is prevented in MMP-12^−/−^ mice and by overexpression of TGF-β1 [69]. Shapiro [47] showed that mice lacking NE are partially (approximately 60%) protected against smoking-induced emphysema. Work by Churg et al. [50] has shown that mice exposed to cigarette smoke had 2–3-fold increases in whole-lung gene expression of MMP-2, MMP-9 and MMP-13 at 6 months, whereas TNFRKO (TNF-α-receptor-knockout mice) mice had no increases, suggesting that TNF-α is responsible for enhanced MMP production after smoke exposure.

Clinical studies have shown that all smokers have a degree of airways inflammation, but that only 20% of smokers develop COPD. This suggests that there are protection and susceptibility factors that are almost certainly genetically controlled. It is well documented that the development of emphysema in cigarette smoke-exposed mice is strain-dependent [43,70]. We have also surveyed a variety of mouse strains and found susceptibility varies with strain, suggesting genetic control of cigarette smoke-induced emphysema [52]. Several inbred strains of mice develop emphysema spontaneously due to genetic abnormalities [43]. In addition, genetic manipulation itself can result in emphysema either spontaneously or during development [43,71,72]. We have shown previously that the introduction of a single amino acid mutation in the kinase Hck causes a spontaneous COPD-like lung pathology [73]. The induced mutation increased Hck activity and caused macrophages to accumulate in the lung, where they released inflammatory cytokines that promoted further inflammation and lung damage through the recruitment of large numbers of protease-producing neutrophils [73]. More recently, using gp130^F/F^ mice homozygous for a subtle knock-in mutation in gp130 that deregulates intracellular signalling by the IL-6 cytokine family, we found that gp130^F/F^ mice spontaneously develop emphysema by age 6 months [74]. Within the IL-6 cytokine family, only IL-6 was significantly up-regulated in the lungs of gp130^F/F^ mice, and the genetic targeting of IL-6 in gp130^F/F^ mice (gp130^F/F^:IL-6^−/−^) prevented emphysema [74]. Acute (4-day) exposure to cigarette smoke further augmented the expression of IL-6 in lungs of gp130^F/F^ mice, and sub-chronic (6-week) exposure to cigarette smoke exacerbated emphysematous and apoptotic changes in the lungs of gp130^F/F^ but not gp130^F/F^:IL-6^−/−^ mice. Therefore the discrete targeting of IL-6 signalling may provide an effective therapeutic strategy against human lung disease.

Airflow obstruction and the loss of lung function with time are characteristic features of human COPD. Classical lung function measurements (e.g. resistance and compliance) are poor indices of the changes that occur in smoke-induced emphysema, even though compliance increases. As a result, there is great interest in developing more useful lung function parameters. Research in this area is focusing on forced deflation manoeuvres, flow–volume loop characteristics and indices of thoracic gas volumes. Just as in human disease, simple measurements of small airways dysfunction in rodents are difficult but not impossible. The most specific function test representative of emphysema is loss of elastic recoil, determined from *P*–*V* (pressure–volume) curves (increased compliance or decreased elastance). Recently, Rinaldi et al. [75] found that emphysema progression in mouse models can be monitored over a prolonged period of time by serial invasive measurements of total lung capacity and compliance within the same animal. Importantly, the pulmonary function parameters obtained were found to be more sensitive than inflammatory and morphological changes in the lung because they picked up differences in lung recoil earlier than the corresponding histological quantifications [75]. Jobse et al. [76] recently demonstrated that *V*/*Q* (ventilation/perfusion) SPECT (single-photon emission computed tomography) imaging can detect lung dysfunction in mice chronically exposed to cigarette smoke before CT (computed tomography) detection of structural changes. Thus *V*/*Q* imaging can detect early changes to the lung caused by cigarette smoke and can provide a non-invasive method for longitudinally studying lung dysfunction in pre-clinical models [76].

## MODELLING ACUTE EXACERBATIONS OF COPD

AECOPD are a common cause of morbidity and mortality in COPD patients and place a large burden on healthcare resources. AECOPD may be prolonged, may accelerate the progression of COPD and have a profound effect on the quality of life [77]. The cellular and molecular mechanisms underlying AECOPD are unclear, but there is an increase in neutrophils and concentrations of IL-6, IL-8, TNF-α and LTB_4_ in sputum during an exacerbation [78,79], and patients who have frequent exacerbations have higher levels of IL-6 and lower concentrations of SLPI, even when COPD is stable [80,81]. There is also an increase in the activation of NF-κB in alveolar macrophages during exacerbations of COPD [82]. Thus exacerbations of COPD appear to be due to further amplification of the inflammatory process.

COPD exacerbations have been associated with a number of aetiological factors, including infection (viral and bacterial). Studies have shown that around half of COPD exacerbations are associated with viral infections and that the majority of these were due to influenza [83], RSV (respiratory syncytial virus) and RV (rhinovirus) [6,8,84]. Respiratory viruses produce longer and more severe exacerbations and have a major impact on healthcare utilization [8,84]. Exacerbations associated with the presence of RV in induced sputum had larger increases in airway IL-6 levels [84], suggesting that viruses increase the severity of airway inflammation at exacerbation. RV can also stimulate mucus production from the airway epithelium and thus potentiate sputum production during a COPD exacerbation [84].

The true incidence of respiratory virus infection in AECOPD had been underestimated previously, since viral culture and serology techniques were used. More recent studies have used PCR to detect viral nucleic acid, which is far more sensitive. It is now clear that at least half of all patients with AECOPD have a respiratory viral infection [6,8]. Given the important role of viruses in COPD, we have developed unique *in vivo* models to investigate the impact of viral infection on cigarette smoke-exposed mice. The advantage of our studies is that we are using live replication-competent viruses rather than replication-deficient adenovirus [85]. Compared with smoke or influenza (H3N1, Mem71 strain) alone, mice exposed to cigarette smoke for 4 days and then influenza had more macrophages, neutrophils and total lymphocytes in BALF at day 3, more macrophages in BALF at day 10, lower net gelatinase activity and increased activity of TIMP-1 in BALF at day 3, altered profiles of key cytokines and CD4^+^ and CD8^+^ T-cells, worse lung pathology and more virus-specific-activated CD8^+^ T-cells in BALF [86]. Mice exposed to smoke before influenza infection had close to 10-fold higher lung virus titres at day 3 than influenza-alone mice, although all mice had cleared virus by day 10, regardless of smoke exposure [86]. Smoke exposure caused temporary weight loss and, when smoking ceased after viral infection, mice exposed to smoke and influenza regained significantly less weight than smoke-alone mice [86]. Therefore, in most respects, smoke exposure worsened the host response to influenza.

Similar findings have been reported by the group of Stamplfi in both acute [87] and chronic [88] cigarette smoke exposure protocols, but using a different strain (C57BL/6) and sex (female) of mice and influenza A virus (H1N1, A/FM/1/47). Using a 4-day cigarette smoke-exposure protocol, they found that smoke-exposed mice had an exacerbated inflammatory response following influenza infection, and that smoke exposure did not compromise viral clearance [87]. However, in the chronic smoking model (3–5 months), cigarette smoke exposure attenuated the airway's inflammatory response to low-dose influenza infection, but there was increased inflammation in smoke-exposed compared with sham-exposed mice after infection with high-dose influenza, despite a similar rate of viral clearance. The heightened inflammatory response was associated with increased expression of TNF-α, IL-6 and type 1 IFN (interferon) in the airway and increased mortality. Importantly, smoke exposure did not interfere with the development of influenza-specific memory responses. Taken together, it is clear from the above studies that cigarette smoke exacerbates the inflammatory response to influenza A virus and that these animal models may be useful in studying how smoke worsens respiratory viral infections.

Live viruses can be difficult to work with and often require special containment facilities. Therefore some groups have used poly(I:C), a synthetic analogue of double-stranded RNA which activates TLR3 (Toll-like receptor 3), to simulate viral infections and to model COPD acute exacerbation-like changes. Kang et al. [89] exposed mice to cigarette smoke for 4 weeks and, during the last 2 weeks of the exposure, the mice were also administered four doses of poly(I:C). Cigarette smoke enhanced parenchymal and airway inflammation and apoptosis induced by poly(I:C). In addition, cigarette smoke and poly(I:C) also induced accelerated emphysema and airway fibrosis. The effects of a combination of cigarette smoke and poly(I:C) were associated with the early induction of type I IFN and IL-18, later induction of IL-12/IL-23 p40 and IFN-γ, and the activation of PKR (double-stranded RNA-dependent protein kinase) and eIF-2α (eukaryotic initiation factor-2α). Importantly, cigarette smoke enhanced the effects of influenza, but no other agonists of innate immunity in a similar fashion. These studies demonstrate that cigarette smoke selectively augments the airway and alveolar inflammatory and remodelling responses induced in the murine lung by poly(I:C) and viruses. More recently, cigarette smoke exposure significantly exacerbated poly(I:C)-induced neutrophilia and airway hyper-responsiveness in mice [90]. Thus, although poly(I:C) may be a useful surrogate to simulate viral infections, key findings should be validated in systems using live replicating influenza viruses.

## MODELLING CO-MORBIDITIES OF COPD

COPD is often associated with co-morbidities that can have an impact on the patient's functional capacity, quality of life, and also increase the risk of hospitalization and mortality in COPD patients [9]. These co-morbidities include skeletal muscle wasting (cachexia), cardiovascular disease, lung cancer, osteoporosis and diabetes [9]. It is currently not clear whether these co-morbidities are independent co-existing conditions (as a result of the advanced age or smoking history of the patient) or a consequence of the patients’ COPD. Nevertheless, COPD patients have a greater risk of cardiovascular disease, and COPD patients are therefore at greater risk of dying from cardiovascular causes. In fact, a number of studies have reported that up to 40% of deaths in COPD patients is attributed to cardiovascular disease [91–93]. Specifically, patients with COPD had a significantly higher risk of congestive heart failure, arrhythmia and acute myocardial infarction [94]. Skeletal muscle wasting is a powerful predictor of mortality in COPD, independent of the lung function impairment [95]. Clinically, rapid deteriorations in lean muscle mass have been described following acute exacerbations of COPD. The disability associated with skeletal muscle wasting is due to both loss of strength and endurance [96–98]. Increased ROS and oxidative stress have been implicated in muscle wasting associated with COPD [99–103]. Post-translational oxidative modifications of quadriceps muscle proteins have been described in smokers and patients with severe COPD, and muscle carbonylation levels were inversely correlated with quadriceps muscle force [104].

Given that co-morbidities have a profound impact on COPD patients, much research has now focused on developing pre-clinical animal models of systemic co-morbidities associated with COPD to determine the mechanisms underlying these conditions and to ultimately identify novel therapeutic options for these patients. A number of studies in mice have shown that cigarette smoke exposure not only leads to pulmonary impairments, but also results in extrapulmonary manifestations frequently observed in COPD. We have shown that mice exposed chronically (4–6 months) to cigarette smoke had increased BALF inflammation, peripheral airspace enlargement, impaired lung function and had reduced body weight, fat mass, hindlimb skeletal muscles mass (gatrocnemius, tibialis anterior and gastrocnemius muscles), grip strength (index of muscle strength) and aerobic endurance [105–111]. Cigarette smoke altered the mRNA expression of a number of genes associated with the regulation of skeletal muscle mass, including IGF-I (insulin-like growth factor-I), atrogin-1 and IL-6 [110,111]. Moreover, the levels of neuropeptide Y, an orexigenic neuropeptide whose activity in the hypothalamic paraventricular nucleus governs appetite, were reduced in cigarette smoke-exposed mice [105]. Gosker et al. [112] have shown that mice exposed to cigarette smoke for 6 months had pulmonary inflammation and emphysema, increased circulating levels of the pro-inflammatory cytokine TNF-α, there was a tendency for the soleus muscle to be lighter after smoke exposure and that the oxidative fibre type IIA proportion was significantly reduced in the soleus muscle. Moreover, muscle oxidative enzyme activity was slightly reduced after smoke exposure, being most prominent for citrate synthase in the soleus and tibialis muscles. Tang et al. [113] also found that mice exposed to cigarette smoke daily for 8 or 16 weeks had elevated serum TNF-α, a loss of body and gastrocnemius muscle complex mass, with rapid soleus fatigue and diminished exercise. Recently, Basic et al. [114] found that, compared with air-exposed mice, skeletal muscles from cigarette smoke-exposed (6 months) mice exhibited significantly enhanced expression of VHL (von Hippel–Lindau tumour suppressor), UBE2D1 (ubiquitin-conjugating enzyme E2D1) and PHD2 (prolyl hydroxylase 2). In contrast, HIF-1α (hypoxia-inducible factor-1α) and VEGF (vascular endothelial growth factor) expression were reduced. Furthermore, reduced muscle fibre cross-sectional area, decreased skeletal muscle capillarization and reduced exercise tolerance were also observed in cigarette smoke-exposed animals [114].

A recent study has explored disease progression while measuring lung function and peripheral skeletal muscle dysfunction (by determining skeletal muscle function and fibre-type distribution) over time in the same animal [75]. The authors found that emphysema progression in mouse models can be monitored over a prolonged period of time by serial invasive measurements of total lung capacity and compliance within the same animal. Importantly, the pulmonary function parameters obtained were found to be more sensitive than inflammatory and morphological changes in the lung because they picked up differences in lung recoil earlier than the corresponding histological quantifications. These data suggest that early emphysema with a loss of elasticity was already present after 3 months of cigarette smoke exposure while using a nose-only exposure system. In this model, muscular changes became apparent only after 6 months, particularly in muscles with a mixed fibre-type composition [75]. Beckett et al. [54] showed that, after 8 weeks of nose-only smoke exposure, mice had chronic lung inflammation, mucus hypersecretion, airway remodelling, emphysema, reduced lung function and decreased quadriceps muscle mass [54]. Thus it is evident from the above animal models that chronic cigarette smoke exposure results in systemic features that closely resemble extrapulmonary manifestations observed in COPD patients, and that these murine models can be used to explore therapeutics aimed at treating skeletal muscle dysfunction observed in human COPD [108].

New animal models with improved clinical relevance are also being developed to investigate the link between COPD and cardiovascular co-morbidities. Recently, it has been shown that chronic cigarette smoke exposure enlarged ventricular end-systolic and diastolic diameters, reduced myocardial and cardiomyocyte contractile function and disrupted intracellular Ca^2+^ homoeostasis, and facilitated fibrosis, apoptosis and mitochondrial damage [115]. Sussan et al. [116] have also shown that mice chronically exposed to cigarette smoke that had chronic lung inflammation and emphysema had pulmonary hypertension and significant impairments to right ventricular diastolic and systolic function and contractility [116]. In addition, Beckett et al. [54] found that hearts from mice exposed to cigarette smoke for 8 weeks that had pulmonary impairments were significantly larger and heavier than air-exposed mice. Moreover, Talukder et al. [117] showed that chronic cigarette smoking causes hypertension, endothelial dysfunction and cardiac remodelling in mice. Of interest is that drugs that have an impact on the cardiovascular system (e.g. statins and angiotensin II blockers) have recently been shown in animal models to protect against cigarette smoke-induced lung inflammation, emphysema and pulmonary hypertension and may therefore provide an important therapeutic benefit for COPD patients [118–120].

## DO PRE-CLINICAL MODELS OF COPD PREDICT THE EFFICACY OF NEW TREATMENTS FOR COPD?

Animal models of COPD have provided valuable insights into the cellular and molecular mechanisms involved in the pathogenesis of COPD. In fact, many of the drugs in clinical development for COPD have been identified from work performed in these models. However, the predictive utility of these models has been questioned, given that fewer than the expected targets identified in animal models have been translated into humans.

### Inhibition of specific inflammatory mediators

Animal models of COPD have recapitulated the increased levels of various inflammatory mediators observed in human COPD, including lipids, cytokines, chemokines and growth factors. As a result, several inhibitors of inflammatory mediators are in development for the treatment of COPD. To date, inhibitors of LTB_4_, TNF-α, IL-1, IL-8 [CXCR2 (CXC chemokine receptor 2) antagonists] and EGF (epidermal growth factor) have produced disappointing results in patients with COPD [121]. Inhibitors of IL-6, IL-17, IL-18, GM-CSF and TGF-β have not been carried out in patients with COPD [121–123].

### Anti-proteases

Animal models of COPD have demonstrated that the serine protease NE plays a key role in the pathogenesis of emphysema as it degrades matrix proteins. NE-knockout mice are protected from cigarette smoke-induced emphysema [124], and cigarette smoke-exposed animals treated with synthetic elastase inhibitors have also revealed the potential anti-inflammatory activity of NE inhibitors [125]. However, although the elastase inhibitor AZD9668 is effective in animal models of COPD, there was no clinical improvement over a 3-month period in COPD patients [126]. Similarly, in a chronic cigarette smoke study in guinea-pigs, the selective MMP-9/MMP-12 inhibitor AZ11557272 reduced pulmonary recruitment of macrophages and neutrophils and was protective against the development of emphysema and small airway remodelling [127]; however, clinical development was stopped for unknown reasons [121].

### PDE4 (phosphodiesterase 4) inhibitors

The pulmonary anti-inflammatory activity of PDE4 inhibitors observed in the animal models of COPD has translated into the clinic. COPD patients treated with the PDE4 inhibitor roflumilast show an improvement in lung function; however, the clinical efficacy of roflumilast is limited by side effects such as nausea, diarrhoea and headaches [121]. In order to overcome these systemic side effects, drug companies have developed potent PDE4 inhibitors that can be delivered by inhalation. However, when UK-500,001 was administered twice daily for 6 weeks to patients with moderate-to-severe COPD, it had no effect on lung function or symptoms [128]. GSK256066 has anti-inflammatory effects in animal models of pulmonary inflammation [129], but further studies are required to confirm the safety profile and to demonstrate clinical efficacy of this compound [130]. Isoenzyme-selective inhibitors have also been explored in animal models of COPD in an effort to overcome side effects. A nebulized antisense oligonucleotide that blocks PDE4B and PDE7A (TPI 1100) inhibits cigarette smoke-induced lung inflammation in mice [131], but a Phase I study was terminated for unspecified reasons [121].

### Kinase inhibitors

The potential therapeutic utility of inhibiting kinases [e.g. p38 MAPK (mitogen-activated protein kinase) and PI3K (phosphoinositide 3-kinase)] in COPD has been supported by data generated in animal models of cigarette smoke-induced lung inflammation. The selective p38α MAPK inhibitor SD-282 inhibited cigarette smoke-induced lung neutrophilia and macrophage recruitment, and decreased tissue mucin staining and phospho-p38 levels in macrophages and epithelial cells [132]. A number of oral p38 MAPK inhibitors have been evaluated in Phase II clinical trials, but these had minimal effects on lung function or sputum neutrophils [121]. Inhaled drugs are in clinical trials, but the results have not yet been reported. An inhaled PI3Kγ and PI3Kδ inhibitor (TG100-115) suppresses lung inflammation in a murine model of cigarette smoke-induced COPD, and clinical studies with inhaled PI3Kδ and PI3Kγ/PI3Kδ dual inhibitors are planned [121].

### Overcoming steroid resistance

It has been proposed that the relative steroid insensitivity of lung inflammation observed in COPD patients is due to the reduction of HDAC (histone deacetylation) activity in the lungs of these patients and that the extent of the reduction in HDAC2 activity correlates with COPD disease status [133]. In support of this, BAL cell influx and emphysema induced by cigarette smoke in animals is not modulated by treatment with corticosteroids [54,130]. Moreover, HDAC2 activity has been shown to be reduced in rodent cigarette smoke models [134], providing a possible link between the steroid insensitivity observed in animal cigarette smoke models and COPD patients. In addition, the steroid-resistant nature of the short-term cigarette smoke model has been used as a means to explore this insensitivity and examine whether it can be restored by pharmacotherapy [135]. These studies have shown that low-dose inhaled theophylline can unlock the resistance. enabling significant anti-inflammatory activity of inhaled steroids to be achieved [135]. Clinical trials with low-dose theophylline combined with corticosteroids are now underway to examine whether the reversal of steroid resistance observed in the cigarette smoke animal model translates to anti-inflammatory activity in the lungs of COPD patients [121,136]. Therefore the steroid-insensitive nature of the animal model of COPD is an important validation of the similarity between the inflammation induced by cigarette smoke in animal models and that observed in COPD patients.

### Antioxidants

Given that oxidative stress is elevated in COPD and that it is a major mechanism that leads to corticosteroid resistance in COPD through reduced activity and expression of HDAC2, animal models of cigarette smoke-induced COPD have explored the therapeutic utility of antioxidant compounds. The antioxidant sulforaphane increases HDAC2 activity and expression and reverses corticosteroid resistance in mice exposed to cigarette smoke and in macrophages from COPD patients [137]. We and others have shown that the antioxidant enzyme Gpx-1 protects against cigarette smoke-induced lung inflammation [53] and emphysema [138] in mice and that the Gpx mimetic ebselen reduces cigarette smoke-induced lung inflammation when administered both prophylactically and therapeutically [53], raising the possibility that Gpx-1 mimetics may have therapeutic potential in COPD [38]. Knockout of the antioxidant response gene Nrf2 (nuclear erythroid-related factor 2) leads to increases in lung inflammation and emphysema in cigarette smoke-exposed mice [139], and an activator of Nrf2 is currently in clinical trial for COPD [121].

## CONCLUSIONS

COPD is a complex inflammatory airway disease characterized by airflow limitation that is not fully reversible. The mechanisms and mediators that drive the induction and progression of chronic inflammation, emphysema and altered lung function are not understood, and this has severely hampered the development of effective treatments for COPD. Current treatments have limited efficacy in inhibiting chronic inflammation, do not reverse the pathology of disease and fail to modify the factors that initiate and drive the long-term progression of disease. Therefore there is a clear need for new therapies that can prevent the induction and progression of COPD. Animal modelling systems that accurately reflect disease pathophysiology continue to be essential for the development of new therapies. The characteristic features of human COPD including neutrophilia, the accumulation of macrophages and T-cells, production of cytokines, chemokines and proteases, oxidative stress, small airway fibrosis/remodelling, mucus hypersecretion, lung dysfunction and the development of emphysema can all be replicated in mice by exposure to cigarette smoke. In addition, new models mimicking acute exacerbations and systemic co-morbidities that more accurately reflect the clinical disease have been developed. However, it is clear that no one animal model is an exact mimic of human COPD and each choice of an animal model has its own benefits and deficiencies. Moreover, the disease they produce is mild, probably equivalent to human GOLD stage 1 or 2 disease, whereas in humans, the majority of morbidity and mortality occurs in patients with GOLD stage 3 or 4 disease. The variability of translatability has led some to suggest that smoke models have limited utility. However, Churg and Wright [16] have proposed that ‘late intervention’ animal models (i.e. starting treatment at 3 months of a 6-month cigarette smoke exposure protocol) may provide a much better indication of therapies that are of benefit in humans. Alternatively, more attention should be directed towards treating patients with GOLD stage 1–2 disease. In recent years, it is becoming evident that COPD patients may have different phenotypes and that there is an incomplete understanding of the molecular, cellular and physiological mechanisms that distinguish sub-populations [140]. Therefore pre-clinical models can only be designed to reflect our current state of knowledge of disease and, if our understanding of the disease we are modelling is inadequate, then these systems will lack the ability to effectively predict drug efficacy. Stevenson et al. [140] have recently proposed that a strategy involving (i) novel bioinformatics methods that can be used to identify animal models that best represent specific patient populations, and (ii) innovative physiological techniques that will improve our ability to discover drugs that can restore the functional capacity of lungs damaged during the course of the disease, may be a way of improving the predictive nature of these models. Finally, animal models utilizing cigarette smoke exposure remain one of the most rigorous means we have to prioritize novel targets for COPD, and it is essential to combine knowledge gained through *in vivo* models, clinical observations and clinical specimens to continually improve animal models so that they remain a valuable tool in the drug discovery process and to ultimately further understand, define and treat COPD.
